# Performance of a 6D Treatment Chair for Patient Positioning in an Upright Posture for Fixed Ion Beam Lines

**DOI:** 10.3389/fonc.2020.00122

**Published:** 2020-02-11

**Authors:** Yinxiangzi Sheng, Jiayao Sun, Weiwei Wang, Brian Stuart, Lin Kong, Jing Gao, Dan You, Xiaodong Wu

**Affiliations:** ^1^Department of Medical Physics, Shanghai Proton and Heavy Ion Center, Shanghai, China; ^2^Shanghai Engineering Research Center of Proton and Heavy Ion Radiation Therapy, Shanghai, China; ^3^Executive Medical Physics Associates, Miami, FL, United States; ^4^Department of Radiation Oncology, Shanghai Proton and Heavy Ion Center, Fudan University Cancer Hospital, Shanghai, China; ^5^Department of Radiation Oncology, Shanghai Proton and Heavy Ion Center, Shanghai, China; ^6^Department of Medical Physics, Shanghai Proton and Heavy Ion Center, Fudan University Cancer Hospital, Shanghai, China

**Keywords:** 6D treatment chair, sitting posture, upright posture, fixed beam line, ion radiotherapy, mechanic accuracy, position alignment

## Abstract

**Purpose:** To evaluate the mechanical accuracy and the robustness of position alignment under x-ray-based image guidance of a treatment chair with six degrees of freedom (6DTC) which was developed for patient treatment in an upright posture at fixed horizontal beam lines in particle (proton, carbon ion, or others) radiotherapy facilities.

**Method and Material:** The positional accuracy including translational and axial rotational accuracy of the 6DTC was evaluated by using a Vicon Motion Capture System (VMCS). Stability of the chair rotation isocenter was determined by a CCD camera with an in-house developed software. The tests were carried out to examine two key motion components of the 6DTC: a floor/rail-mount 360°-rotating platform and a 6-degree-of-freedom (6DOF) platform. The measurement results were compared to that of a commercial clinical robot couch. The accuracy of position alignment, simulating the actual clinical protocol, through an Image-guided Radiation Therapy (IGRT) system was studied at the pre-treatment position and beam specific treatment position.

**Results:** The translational accuracy was 0.12 mm (SD 0.07 mm) for the 6DOF platform. The rotational accuracy was 0.04° (SD 0.03°) and 0.02° (SD 0.02°) for the 6DOF platform and the 360° -rotating platform, respectively. The displacement between the chair rotation center and the room isocenter center was no more than 0.18 mm in all three rotational axes. Combined with an x-ray-based IGRT system, the treatment alignment test with a rigid phantom yielded a total positional accuracy of 0.23 mm (SD 0.17 mm) and 0.14° (SD 0.14°) at treatment position.

**Conclusions:** On the basis of the rigid phantom study, the 6DTC showed comparable accuracy to the robot treatment couch. Combining with the IGRT, the 6DTC can provide position alignment with submillimeter accuracy for rigid phantom in upright posture.

## Introduction

Charged particles such as protons or carbon ions deposit most of their energy at the Bragg peak area when transport in media, little at the entrance and almost zero at the distal end. Therefore, charged particle therapy (CPT) could produce highly conformal dose distributions in the target volume with markedly lower dose to the normal tissues (1–3). Heavy ion radiotherapy provides not only dosimetric, but also biological advantages compared with photon radiotherapy (4, 5).

Clinical experience shows that flexibility in the selection of beam number and directions could greatly help in achieving robust plans with high dose conformality (6). In conventional or traditional radiotherapy, most patients are positioned on a treatment couch, treated by gantry-mounted photon/electron linear accelerators. To achieve the same degree of freedom in the selection of beam angles, most of proton centers in operation worldwide are equipped with at least one gantry (7). However, proton and especially carbon ion gantries are enormously heavy and expensive (8). The first carbon gantry in operation is in HIT (Heidelberg Ion Beam Therapy Center) with a rotating mass weighing more than 600 tons (9). Given the physical advantage of the Bragg peak, the range of beam entry directions for charged particle beams might not be as crucial as that of photon beams. This reasoning led to the design and implementation of treatment rooms with fixed beam lines.

In most of treatment rooms with fixed beam lines, treatment couch is still a standard configuration for patient positioning. Although this is satisfactory in a wide range of clinical indications, additional flexibility in beam angle selection is found desirable in some selective disease sites, such as head and neck. An alternative is to have patients sit in an upright treatment position in a treatment chair. Patient treatment in an upright posture was first reported in 1960s (10). Miller et al. (11) constructed a chair that could permit isocentric alignment with patients in an upright sitting position around the vertical rotational axis. Rachel et al. (12) constructed a chair that allowed for three-dimensional imaging and treatment delivery. The reported experience showed that the reproducibility of inter- and intra-fraction displacement for patients in an upright posture was comparable to that reported for patients in the supine lying position. In 2018, Balakin et al. (13) described the clinical use of an immobilization system in seated position for proton radiotherapy, however, no performance characteristics of the chair was reported. With the objective of maximizing the selection of the incident angles of beams, especially along anterior-posterior direction of patients in fixed beam lines, a treatment chair with six degrees of freedom (6DTC) was recently designed and constructed in our facility, intended for but not limited to the fixed horizontal beam line of proton and carbon ion radiotherapy.

Before the clinical usage of this 6DTC, it is necessary to verify the system's performance. In this study, we first evaluated the performance of the 6DTC in terms of its motion characteristics and mechanical accuracy in all six degrees of freedom. The results were compared with the data from a commercial robot treatment couch, which has been clinically used in our facility. Subsequently we proposed the position alignment procedures for the 6DTC in combination with the Image-guided Radiation Therapy (IGRT) system. The IGRT system integrated in the treatment room for high precision patient positioning and setup verification, initially configured for treatment with the robot couch, was modified to be compatible with the 6DTC. The initial results of the position alignment accuracy of the 6DTC with a rigid phantom were reported. In addition, the considerations for the clinical implementation of the 6DTC were briefly discussed.

## Methods and Materials

### Compositions of the 6DTC

**Figure 1:**
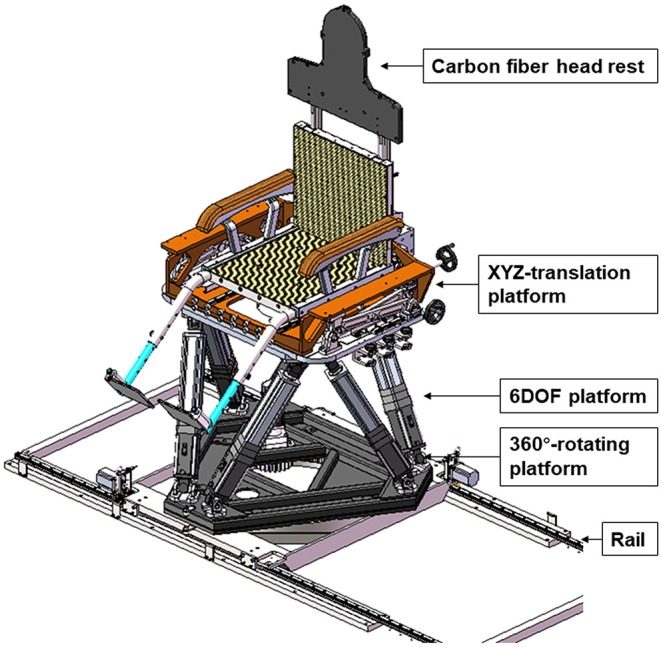
3D model of 6DTC and its components.

The 6DTC consists of a floor/rail-mount 360°-rotating platform, a 6-degree-of-freedom (6DOF) Stewart hexapod platform (14), a XYZ-translation platform, and a seat with a carbon fiber head rest. Figure 1 shows the 3D model of the 6DTC with all composition modules.

The 360°-rotating platform provides the rotation range of ±180° around the vertical axis, with an increment of 0.1°.

The 6DOF platform was assembled on top of the 360°-rotating platform. The designed motion range of the 6DOF platform is ±200 mm in *x* and *y* directions and ±120 mm in *z* direction, and ±20° rotating range around all three axes. The minimum movement steps of the 6DOF platform are 0.1 mm and 0.1°.

The XYZ-translation platform was mounted on top of the 6DOF platform, and was intended for providing additional translation motion range. The designed translation motion range of the XYZ- translation platform is ±250 mm in *x, y*, and *z* directions. This platform can only be maneuvered manually.

The carbon fiber headrest was designed to be compatible with conventional patient immobilization devices such as alpha-cradles and head thermoplastic masks. It can be used for patient immobilization in both lying and sitting treatment postures.

The whole 6DTC is placed on a rail such that it can be moved between treatment position and parking position. The 6DTC in parking position will not cause interference when the robot couch is used for treatment.

The additional details of the design and the composition of the 6DTC will be described in another research paper.

### Coordinate System and Position/Motion Monitoring and Measurement

**Figure 2:**
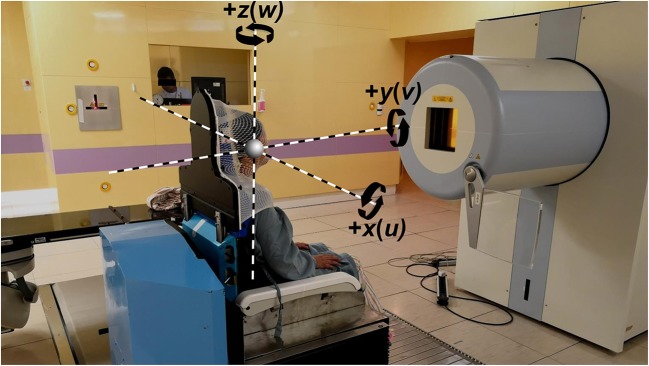
Coordinate system indicating the x, y, and z translational axis and rotational axis, with the 6DTC in the pre-treatment position in front of the beam nozzle.

The coordinate system and movement of the 6DTC follow the IEC (International Electrotechnical Commission) convention, consistent with what is being currently used in our facility (Figure 2).

A motion capturing system by Vicon (VMCS), V16 (Vicon Motion Systems, LA, USA), with eight motion capturing cameras mounted on the ceiling was used to monitor the position and movement of the 6DTC and the robot couch, with a set of six reflective position/motion markers (spherical, 14 mm in diameter) attached to the 6DTC or to the robot couch. After calibration, the motion capturing system is capable of detecting deformation and displacement with accuracy better than 0.1° and 0.1 mm (15–17).

In addition to measuring and analyzing the translational and rotational movement by the VMCS, the stability of the rotation isocenter, i.e., the origin of the 6DTC coordinate system, was examined by both VMCS and a high-resolution CCD camera with an in-house developed software. All the measurements were performed with 40 kg weight on the top of the 6DTC or on the robotic couch.

### Translational Accuracy

Both the 6DOF platform and the XYZ-translation platform can provide patient translation movement in *x*(lateral), *y*(longitudinal), and *z*(vertical) directions. Following initial seating of patients on the 6DTC, XYZ-translation platform (manually maneuvered) will be locked; the subsequently interactive patient position movement for alignment will rely only on the 6DOF platform. Therefore, the final translational accuracy for alignment is ultimately determined by the 6DOF platform. The accuracy of translational movement was measured by VMCS with the 6DTC excursion sequences of ±5, ±10, ±15, and ±20 cm in x and y directions, and ±5, ±10, and ±12 cm in z direction, respectively. The standard deviation was derived from ten repeated measurements. The same setup was used for the tests on the robot couch.

### Rotational Accuracy

The rotational accuracy was also measured and evaluated by using the VMCS. The 6DOF platform can rotate the 6DTC around the *x, y*, and *z* axes, while the 360°-rotating platform provides only rotation around the *z* axis. The rotation testing sequences for the 6DOF platform around the x, y, and z axis (also referred to as the roll, pitch, and yaw direction rotation) was from −20° to +20° with an increment of 5°. The rotation testing sequences for the 360°-rotating platform was from −180° to 170° with an increment of 10°.

The rotational accuracy of the robot couch was measured by using the rotation sequences: −7° to 7°, −10° to 10° and −100° to 100° in pitch, roll, and yaw direction respectively. The difference in angles is due to the movement restrictions of the robot couch.

### Stability of the Chair Isocenter

To accurately carry out patient position alignment by any patient positioning device, couches or chairs alike, all movements, translation, or rotation are required to center consistently on a reference point that is the origin of the coordinate system and the isocenter of the treatment room. In other words, the coordinate system of the positioning device should precisely coincide with the room treatment coordinate system. This can be most effectively verified by checking the eccentricities of rotation around each axis.

The rotational eccentricity of the 360°-rotating platform will reflect on the displacements of the chair rotation center from the room isocenter, indicated by the room lasers, in x (transverse), y (longitudinal), and z (axial) directions (transverse and longitudinal are also classified together as radial). The measurement and evaluation of the isocenter displacements in x and y direction (radial) were carried out using the high-resolution CCD camera and a coordinate plane graph paper attached to the 6DTC. The displacement in axial, or z direction was carried out using the VMCS. The same is applied to the measurement for the robot couch. The zero position was defined as, and set with the 6DTC and the head of robot couch directly facing the beam nozzle (0° in Cartesian coordinate system).

When measuring the radial (x, and y) isocenter displacement, one of the crossing grids on the graph paper was carefully moved to align with the treatment room laser crosshair and was defined as the marked origin. The deviation or displacement between the marked origin and the laser crosshair was observed through the CCD camera after rotating the 360°-rotating platform through a specific angle. An in-house software was used to measure the displacement between the laser crosshair and the marked origin on the graph paper.

As the 360°-rotating platform is the foundation of the chair, its rotation eccentricity (around Z axis) manifested by the isocenter displacements will affect all subsequent movements by the 6DOF platform. To overcome this, a special function was built into the 6DOF platform to correct for such displacement, and was accomplished by asserting a multi-segment linear calibrating function derived from measuring the isocenter displacement (only in radial directions) of the 360°-rotating platform for full revolution in 10° increment (the details of this measurement is described in Appendix A in Supplementary Material).

To test the effectiveness of this isocenter correction function of the 6DOF platform, the 6DTC was rotated from −180° to 170° in an increment of 10° by the 360°-rotating platform, stopping to record at each increment. The isocenter displacement was evaluated with and without applying the calibration correction of the 6DOF platform. The tests were performed in both clockwise (CW) and counterclockwise (CCW) directions, and repeated 11 times. The isocenter displacement due to rotation was also examined for the robot couch with rotation angles from −100° to 100° with an increment of 10° and with three repeated measurements.

### Accuracy of Position Alignment of 6DTC in Combination With the IGRT System

Parallel to the treatment chair project, a treatment planning module for upright orientation was developed and integrated into the current treatment planning system. The new chair treatment planning module converts images (CT, MRI, etc.) acquired in conventional lying position to vertical upright orientation and allows beam selections and dose optimization according to the 6DTC geometry.

In order to evaluate the overall accuracy and consistency of the 6DTC in terms of patient alignment and position correction, a set of tests have been designed and carried out with an anthropomorphic head phantom (PBU-60, KYOTO KAGAKU, Japan). The Siemen IGRT system was utilized to provide orthogonal KV images and the position correction vectors through the gray value-based registration algorithm. The Siemens IGRT system is part of the integrated delivery system used clinically in our facility and has demonstrated the ability to achieve submillimeter accuracy through 3D/2D image registration (18).

The phantom was immobilized, simulating the sitting treatment posture by using the 6DTC headrest, and was affixed in an alpha-cradle module with a head thermoplastic mask locked down from the top of the face. A computed tomography (CT) scan with 1.5 mm slice thickness was acquired for the phantom in lying position. The CT images were imported into Chair Treatment Planning Module. According to the testing protocol, a series of chair treatment plans with different chair orientations were generated (from −180° to 120° in a 60° interval). Digitally reconstructed radiographs (DRRs) were generated with each plan for the subsequent image-guided “patient” alignment.

The alignment procedure is as follows: the phantom was first setup on the 6DTC at pre-treatment position (6DTC facing the nozzle as shown in Figure 2) with a randomly introduced translational and rotational deviation ranging from −2.31 cm/−1.10° to 2.81 cm/2.00° in all directions. After the acquisition of the first pair of orthogonal kV x-ray images, the positional error (three translation shifts *x*_*p*_, *y*_*p*_, and *z*_*p*_ and three rotation shifts *u*_*p*_, *v*_*p*_, and *w*_*p*_) associated with the pre-treatment position (PE-P) was obtained by using double automatic registration between the KV images and the reference images (DRRs) with the gray value based image automatic matching algorithm. The 6DTC was then moved to the treatment position (different orientations were used every day from Monday to Saturday from −180° to 120° in a 60° interval according to the treatment plans). Subsequently second pair of orthogonal KV images were taken and registered with the DRRs, the positional errors (three translation errors *x*_*t*_, *y*_*t*_, and *z*_*t*_ and three rotation errors *u*_*t*_, *v*_*t*_, and *w*_*t*_) at the treatment position (PE-T) were obtained, and the repositioning of the 6DTC by the correction values (errors) was carried out from inside or outside of the treatment room. Finally, the residue positional error (three translation errors *x*_*r*_, *y*_*r*_, and *z*_*r*_ and three rotation errors *u*_*r*_, *v*_*r*_, and *w*_*r*_) at the treatment position (RPE-T) was obtained by performing the third orthogonal KV image acquisition and automatic matching with the DRRs. The procedure or protocol described above has been implemented clinically and is performed daily with a phantom as the quality assurance (QA) for the 6DTC prior to clinical operation.

The position deviation between PE-T and PE-P represents the positional errors introduced by both the 360° -rotating platform and the 6DOF platform of the 6DTC. The position deviation between RPE-T and PE-T is the position errors introduced by the 6DOF platform alone, and also represents the final accuracy of the 6DTC per afore described alignment protocol.

## Results

### Positional Accuracy and Reproducibility

The positional accuracy was defined as the mean absolute deviations between the intended position and the measured position. The translational accuracy of the 6DOF platform of the 6DTC and the robot couch in three directions are shown in Table 1. The mean absolute translational error was 0.12 mm (Standard Deviation SD 0.07 mm) and 0.08 mm (SD 0.06 mm) in all directions for the 6DTC and the robot couch, respectively. The maximum absolute translational deviation observed was 0.35 mm (for −150 mm excursion in longitudinal direction) and 0.23 mm (for +150 mm excursion in lateral direction) for the 6DTC and the robot couch, respectively.

**Table 1:** Translational accuracy of the 6DTC by using 6DOF platform and the robot couch in lateral, longitudinal, and vertical directions.

**Translational displacement (mm)**		**Lateral error (mm)**				**Longitudinal error (mm)**				**Vertical error (mm)**			
		**6DTC**		**Robot couch**		**6DTC**		**Robot couch**		**6DTC**		**Robot couch**	
**X/Y**	**Z**	**Mean (ABS)**	**SD**	**Mean (ABS)**	**SD**	**Mean (ABS)**	**SD**	**Mean (ABS)**	**SD**	**Mean (ABS)**	**SD**	**Mean (ABS)**	**SD**
−200	–	0.25	0.02	0.17	0.02	0.25	0.02	0.05	0.02	–	–	–	–
−150	−120	0.12	0.01	0.15	0.01	0.26	0.08	0.06	0.02	0.15	0.02	0.08	0.02
−100	−100	0.03	0.02	0.00	0.02	0.12	0.07	0.06	0.02	0.15	0.03	0.09	0.02
−50	−50	0.11	0.02	0.10	0.01	0.12	0.06	0.08	0.02	0.08	0.02	0.03	0.02
50	50	0.12	0.02	0.00	0.01	0.07	0.03	0.01	0.01	0.10	0.03	0.06	0.01
100	100	0.10	0.02	0.10	0.01	0.09	0.03	0.08	0.02	0.12	0.02	0.12	0.01
150	120	0.10	0.03	0.21	0.01	0.15	0.07	0.09	0.02	0.17	0.02	0.18	0.01
200	–	0.04	0.01	0.01	0.01	0.06	0.02	0.09	0.01	–	–	–	–

A relatively larger standard deviation was observed for the 6DTC translational movement in the longitudinal direction especially for the −150 mm excursion, with the mean value of −150.26 mm (SD 0.08 mm). Moving the 6DTC to position −150 mm by 6DOF platform, we found a deviation of 0.15 mm starting from position −100 mm and from position −200 mm. This phenomenon was also observed in the excursion of −100 and −50 mm of the 6DTC, but not in the movement of robot couch.

The rotational accuracy of the 6DOF platform and the robot couch in three directions is shown in Table 2. The mean absolute rotational error was 0.04° (SD 0.03°) and 0.01° (SD 0.01°) around all axes for the 6DOF platform and the robot couch, respectively. The maximum absolute rotational error was 0.10° and 0.03° for the 6DOF platform and the robot couch, respectively.

**Table 2:** Rotational accuracy of the 6DTC by using 6DOF platform and the robot couch in pitch, roll, and yaw directions.

**Rotational angle (°)**		**Pitch error (°)**				**Roll error (°)**				**Yaw error (°)**			
		**6DTC**		**Robot couch**		**6DTC**		**Robot couch**		**6DTC**		**Robot couch**	
**6DTC**	**Robot couch**	**Mean (ABS)**	**SD**	**Mean (ABS)**	**SD**	**Mean (ABS)**	**SD**	**Mean (ABS)**	**SD**	**Mean (ABS)**	**SD**	**Mean (ABS)**	**SD**
−20	–	0.04	0.00	–	–	0.02	0.00	–	–	0.01	0.00	0.00	0.01
−15	–	0.02	0.01	–	–	0.04	0.01	–	–	0.01	0.00	–	–
−10	−7	0.06	0.01	0.00	0.00	0.05	0.01	0.03	0.00	0.01	0.00	0.00	0.01
−5	−5	0.03	0.01	0.00	0.00	0.01	0.01	0.03	0.00	0.02	0.01	–	–
5	5	0.01	0.01	0.00	0.00	0.02	0.01	0.03	0.00	0.03	0.01	–	–
10	7	0.03	0.01	0.00	0.00	0.01	0.01	0.03	0.00	0.03	0.01	0.00	0.01
15	–	0.08	0.01	–	–	0.04	0.00	–	–	0.06	0.01	–	–
20	–	0.09	0.01	–	–	0.01	0.00	–	–	0.01	0.01	0.01	0.01

**Figure 3:**
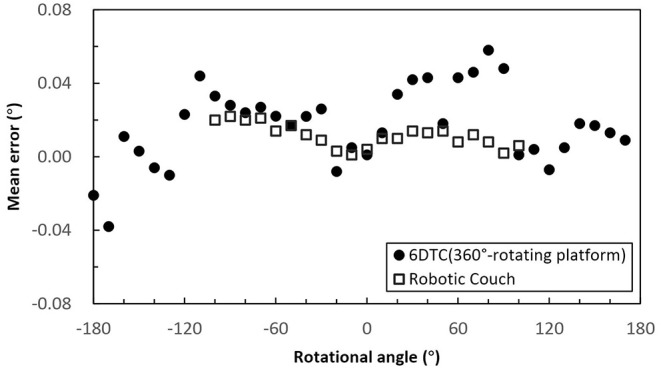
Mean rotational error of the 6DTC by using the 360° -rotating platform and the robot couch in yaw direction.

The rotational accuracy of the 6DTC by using the 360° -rotating platform is shown in Figure 3. The mean absolute rotational error was 0.02° (SD 0.02°) with the maximum absolute rotational error of 0.10°.

### Stability of Chair Rotation Isocenter

**Figure 4:**
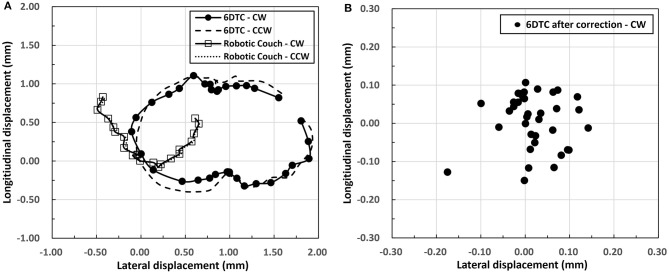
**(A)** Radial displacements of rotation isocenter for the 6DTC and the robot couch. The circles along the solid curve represent CW rotation for 6DTC. The dashed curve represents CCW rotation for the 6DTC. The squares hollow along the solid curve represent CW rotation for robot couch. The dotted curve represents CCW rotation for robot couch. **(B)** Displacements of chair rotation isocenter after correction by the 6DOF platform.

The plot of the radial errors (displacements) of rotation isocenter of the 6DTC and the robot couch measured using the high-resolution CCD cameral is shown in Figure 4A. The plotted points represent the average of the lateral (x) and longitudinal (y) shifts at each angle for both the 6DTC and the robot couch, with both Clockwise (CW) and Counterclockwise (CCW) rotations. The mean absolute error of all measured points at a given rotation angle from all runs for the 6DTC before 6DOF platform correction were 0.76 mm (SD 0.47 mm) and 0.40 mm (SD 0.28 mm) in the lateral and longitudinal directions, respectively. The measurement results were 0.34 mm (SD 0.19 mm) and 0.30 mm (SD 0.25 mm) for the robot couch in the lateral and longitudinal directions, respectively. The points with the largest deviations from the mean value in lateral and longitudinal directions are −0.70 mm on the CW path at −180°, and −0.57 mm on the CW path at 120° for the rotations of the 6DTC.

Figure 4B shows the chair rotation isocenter radial displacement after correction by the 6DOF platform. Only CW rotation results were shown in this figure, as the results from CCW rotation fell in the same range. The mean absolute radial error from all runs were 0.05 mm (SD 0.04 mm) and 0.06 mm (SD 0.04 mm) in the lateral and longitudinal directions, with the maximum absolute displacement no more than 0.18 mm for all rotation angles measured.

**Figure 5:**
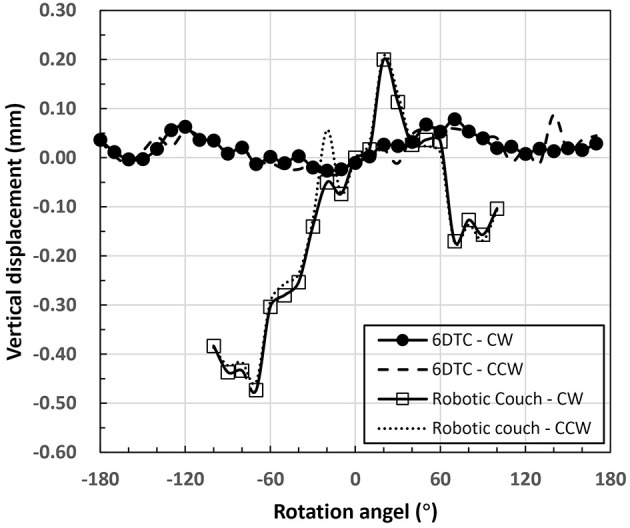
Axis rotation isocenter displacements of the 6DTC and the robot couch.

Figure 5 shows the axial (z) rotation isocenter displacements of all rotation angles for the 6DTC and the robot couch measured by the VCMS. The plotted points indicate the average of measurements for all angles. The mean absolute displacements were 0.03 mm (SD 0.02 mm) and 0.18 mm (SD 0.14 mm) for the 6DTC and the robot couch, respectively. The greatest absolute displacements were 0.11 mm and 0.48 mm for the 6DTC and the robot couch, respectively.

The difference between CW and CCW directions in the mean isocenter displacements for all the measurement angles in all axes was no more than 0.15 and 0.03 mm for the 6DTC and the robot couch, respectively.

### Position Alignment With IGRT

The alignment tests were repeated forty times with a random positional “error” introduced in each pre-treatment position. The position deviations between PE-T and PE-P and between RPE-T and PE-T are shown in Table 3.

**Table 3:** The position deviation or offset.

	**Translational errors[mm]**						**Rotational errors[°]**					
	**x**		**y**		**z**		**u**		**v**		**w**	
	**Mean (ABS)**	**SD**	**Mean (ABS)**	**SD**	**Mean (ABS)**	**SD**	**Mean (ABS)**	**SD**	**Mean (ABS)**	**SD**	**Mean (ABS)**	**SD**
Deviation between PE-T and PE-P	0.63	0.43	0.58	0.31	0.06	0.08	0.10	0.12	0.09	0.10	0.04	0.08
Deviation between RPE-T and PE-T	0.23	0.17	0.10	0.14	0.09	0.09	0.14	0.14	0.00	0.00	0.06	0.09

*The offsets between PE-T and PE-P represent the residual errors after the first move from pre-treatment position to treatment position. The offsets between RPE-T and PE-T represent the final residual errors after the second position correction performed by the 6DTC*.

The mean absolute position deviations between PE-T and PE-P were 0.63 mm (SD 0.43 mm), 0.58 mm (SD 0.31 mm), 0.06 mm (SD 0.08 mm), 0.10° (SD 0.12°), 0.09° (SD 0.10°), and 0.04° (SD 0.08°) in the x, y, z, u, v, and w directions, respectively. The maximum deviation over all the measurements was −1.6 mm translational in *x* and 0.4° rotational around *u*.

The mean absolute position deviations between RPE-T and PE-T were <0.23 mm for translational and 0.14° for rotational. The reduced mean absolute value indicates the further improved positioning accuracy after the final position correction. The maximum standard deviation of 0.17 mm and 0.14° for translation and rotation errors represent very small random uncertainties. Translation correction vectors in z direction shows the lowest offset among all three set of experiments.

## Discussion

In this study, the performance of the 6DTC was investigated comprehensively. The results show that the 6DOF platform and the 360°-rotation platform could together make the 6DTC a patient positioner with sufficient precision for proton and carbon ion radiotherapy.

The results in Tables 1, 2 and Figure 4 show that the total mechanical accuracy of the 6DOF platform in terms of the mean absolute errors were always <0.26 mm and 0.09°. Meanwhile, the small standard deviation of <0.03 mm and 0.01°, indicate its excellent stability.

Various alternative positioning systems have been described in the literature. Meyer et al. (19) reported the mechanical positional accuracy of a hexapod robot treatment table, with the translational positioning accuracy ranging from 0.10 to 0.20 mm in the excursion of 5 cm, and the rotational accuracy ranging from 0.10° to 0.20°. Takakura et al. (20) reported the positional accuracy and reproducibility of a 6D robot couch, with the mean positional error and the standard deviation (SD) being 0.07 ± 0.22 mm and −0.05° ± 0.14° for translation and rotation, respectively. In comparison, the relatively larger mean value of errors and smaller SD from our measurement is due to the fact that we have used mean absolute deviations to represent the position accuracy of the 6DTC and the measurement were based on the VMCS directly instead of the x-ray system with the later being more prone to introducing uncertainties. For a more direct comparison, we measured the positional accuracy of the robot couch in our facility applying the same measurement technique. Results showed 0.21 mm and 0.03° for the translational and rotational accuracy, respectively. The relatively smaller rotational error for the robot couch may be due to the smaller rotation excursion of 7° (internal restriction), while for the 6DOF platform the rotation excursion was 20°. Compared to the data described in the literature and that measured on the commercial robot couch used in our facility, the 6DTC has demonstrated comparable mechanical accuracy and consistency.

It has been well-understood that any misalignment of the rotation isocenter could lead to inaccuracies in the treatment setup. In photon-based stereotactic radiosurgery, the isocentric errors are usually kept under 0.50 mm (21). Moyers et al. (22) reported their evaluation of the Loma Linda University proton treatment system and showed the competitive isocentric accuracy of 0.2 mm or better. For the 6DTC, the accuracy of the mechanical rotation isocenter was found to be better than 0.18 mm in the full applicable range. It has also been noted that the chair isocenter in axial (vertical) direction is more stable than the robot couch and those reported in literature. The absolute error in vertical movement was found to be 0.03 mm (SD 0.02 mm) and 0.18 mm (SD 0.02 mm) for the 6DTC and the robot couch in our center, respectively. This difference may be due mainly to the fact that the center of gravity is largely aligned vertically with the base of the 6DTC while for the robot couch it is distal to the base.

**Figure 6:**
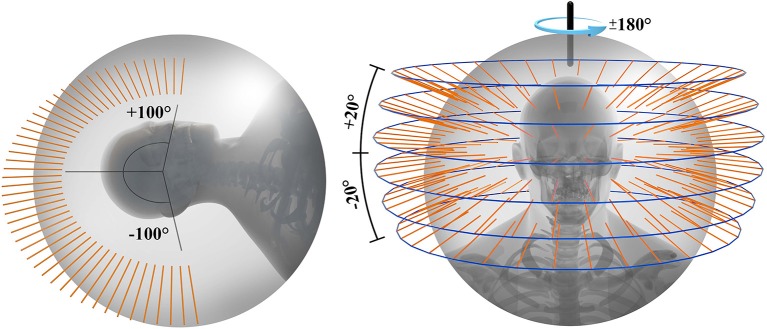
Available beam orientations for fixed ion beam line using robot couch **(left)** and 6DTC **(right)**.

Non-coplanar-beam configuration has been frequently used in conventional radiotherapy. Clinical results have shown reduction of normal tissue volume exposed to low and intermediate dose and subsequent decrease in side effects (23, 24). The design of the 6DTC was specific for the carbon ion or proton therapy with fixed horizontal beam lines, without which treatment angles could only rely on the limited couch rotations. As shown in Figure 6, the 6DTC could extend the angular range, and therefore provide a solution to use non-coplanar radiotherapy with carbon and proton ion beams when only fixed beam lines are available. However, to choose an optimal set of beam orientations for clinical use is challenging, in the context of accuracy of patient immobilization, treatment efficiency, and ion beam range uncertainties, etc. Study on this new endeavor will be reported in a separate paper.

Building upon the mechanical accuracy, the 6DTC has been fully integrated with the IGRT system and hence warrant its clinical applicability. Based on the phantom measurement, combined with the IGRT system, the 6DTC has shown its capability of providing overall position correction accuracy with 0.23 mm/0.14° of setup uncertainty.

Keep in mind that the accuracy test presented herein was conducted with a rigid phantom. In actual clinical implementation, deformations of bony structures and soft tissues are anticipated due to the influence of gravity when the patient changes the posture from lying to upright. Balakin et al. (13) have reported up to 3–4 mm internal movement of patient in the thermoplastic masks during the proton beam irradiation in seated position. Additionally, the patient setup uncertainty in particle therapy is well-recognized as a major contributing factor to the particle beams' range uncertainties (25). Therefore, one should exercise additional consideration when deciding the treatment margin to properly account for all sources of uncertainties. These compounded uncertainties also make the image-guided alignment with either orthogonal x-ray images or in-room-CT indispensible.

## Conclusion

Performance of the treatment chair with 6 degrees of freedom for ion beam radiotherapy in an upright posture is reported in this study and compared with a commercially certified robot treatment couch, in terms of the translational and rotational position accuracy. The abilities for patient position correction with the 6DTC were also evaluated. The mechanical accuracy was shown to be 0.12 mm (SD 0.07 mm) and 0.04° (SD 0.03°), comparable to the robot couch. Combining with the IGRT, the 6DTC can provide position alignment with sub-millimeter accuracy for rigid phantom in upright posture.

## Data Availability Statement

The datasets generated for this study are available on request to the corresponding author.

## Author Contributions

XW and YS contributed conception and design of the study. YS and JS organized the database. JS, WW, and DY performed the statistical analysis. YS wrote the first draft of the manuscript. WW, BS, LK, JG, and XW wrote sections of the manuscript. All authors contributed to manuscript revision, read and approved the submitted version.

### Conflict of Interest

The authors declare that the research was conducted in the absence of any commercial or financial relationships that could be construed as a potential conflict of interest.
