# Supplementary material for: Performance of a 6D Treatment Chair for Patient Positioning in an Upright Posture for Fixed Ion Beam Lines
Source: Front Oncol. 2020 Feb 11;10:122. doi: 10.3389/fonc.2020.00122 (PMC7026365; doi:10.3389/fonc.2020.00122)
Supplement: Supplementary file 1 [file Table_1.DOCX]

**Supplementary material**

**A: The determination of radial shift of chair rotation isocenter by using CCD camera-based in house developed software.**

The measurement of mechanical rotation isocenter in lateral and longitudinal direction (radial) cannot be easily detected by using VCMS, because the coordinates defined in VCMS are based on the reflective markers 14 mm in diameter. It is not feasible to align the center of the reflective ball to coincide with the laser crosshair within 0.1 mm accuracy. Therefore, the high-resolution CCD camera with coordinate plane graph paper has been used to detect the radial shift of mechanical rotation isocenter. As is shown in figure A.1, the graph paper was first aligned to make the marked origin coincide with the room laser; the 6DTC was then rotated around the axial axis (Z). After calibrating the pixel to mm ratio, the shift between the laser center and the chair rotation center (marked origin) can be obtained.


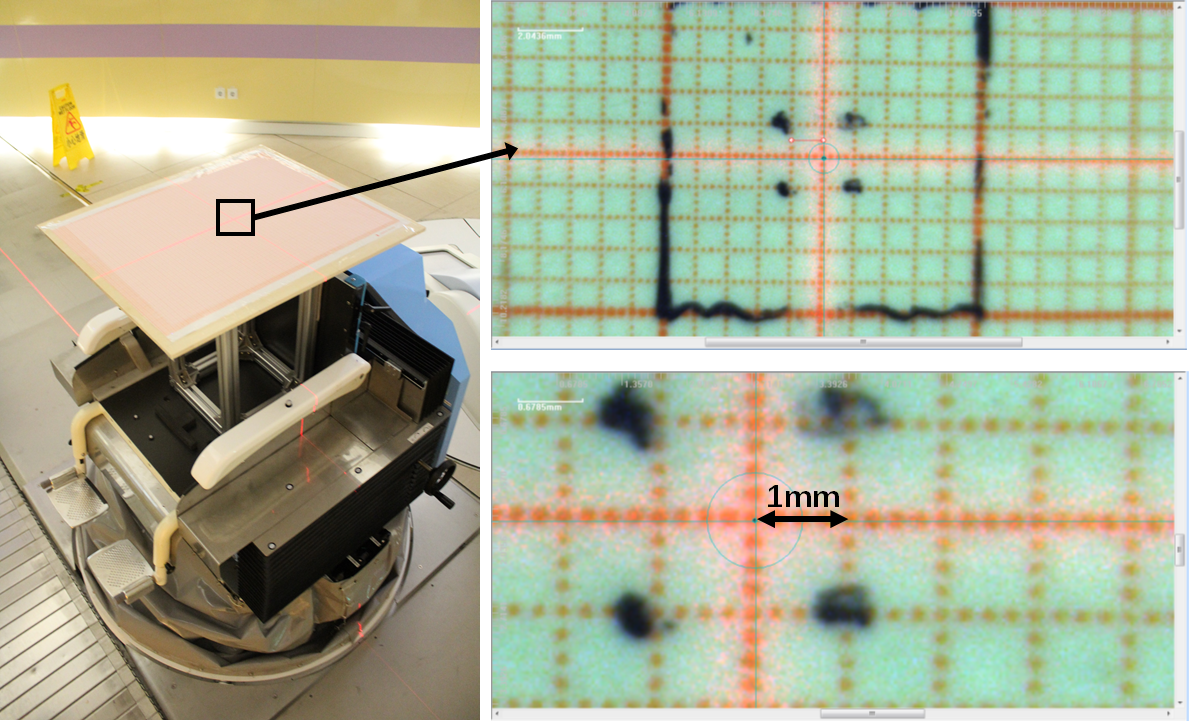


Figure A.1 The measurement of radial isocenter shift by CCD camera and coordinate plane graph paper after calibration (upper right), and an example to align the graph paper to coincide with the laser crosshair (bottom right).
